# Impact of Medical Care on Symptomatic Drug Consumption and Quality of Life in Headache: A One-Year Population Study

**DOI:** 10.3389/fneur.2019.00629

**Published:** 2019-06-18

**Authors:** Matteo Cotta Ramusino, Ilaria De Cillis, Alfredo Costa, Fabio Antonaci

**Affiliations:** ^1^IRCCS Mondino Foundation, Pavia, Italy; ^2^Department of Brain and Behavioral Sciences, University of Pavia, Pavia, Italy

**Keywords:** chronic headache, medical overuse headache, symptomatic drug, epidemiology, medical care

## Abstract

**Background:** Chronic headache is one of the most common pain conditions, often leading to symptomatic drug overuse. The aim of this study was to provide data on symptomatic drug consumption in an Italian outpatient population and to describe how the clinical picture of headache may change after headache experts take charge of the care of affected individuals.

**Methods:** A total of 199 adults complaining of chronic headache were recruited through 32 pharmacies in the Pavia health district. Participants underwent four evaluations: a baseline assessment (T0) and three follow-up evaluations performed by a neurologist at 3, 6, and 12 months (T3, T6, and T12, respectively). On each occasion, they underwent a complete neurological assessment and received therapeutic adjustments to achieve better management of their headache.

**Results:** On the basis of a preliminary telephone interview, the prevalence rates of chronic headache and medication overuse headache (MOH) were 16 and 12%, respectively. At 12 months of follow-up, we observed a significant decrease in the frequency of attacks (T0: 9 ± 9/month vs. T12: 2 ± 2/month; *p* < 0.001), in the number of days/month with headache (T0: 11 ± 9 vs. T12: 4 ± 4; *p* < 0.001) and in single attack duration (T0: 34 ± 30 h vs. T12: 10 ± 19 h; *p* < 0.001). Careful headache management resulted in a significant decrease in analgesic consumption (T0: 12 ± 16 vs. T12: 4 ± 6 doses/month; *p* = 0.014) and a significant increase in quality of life, measured using the Migraine Disability Assessment Scale (MIDAS) and Headache Under-Response to Treatment (HURT) scales (*p* < 0.001).

**Conclusions:** Headache management by a specialist is more effective than self-treatment, resulting in an overall benefit for headache patients.

## Introduction

Headache is a common pain condition, responsible for varying degrees of disability depending on the frequency, duration and intensity of the attacks. The chronic forms, which in most cases have evolved from migraine and tension-type headache, are associated with higher levels of healthcare demand and drug consumption (1).

Chronic headache (CH), defined as headache occurring on at least 15 days per month, has a variable prevalence worldwide: 1.0–3.9% in the Asia-Pacific region (2), 3.7% in the Netherlands (3), 5% in Central/South America and only 1.7% in Africa (4), and it appears to be inversely related to socioeconomic position and physical and mental health status (5). The high disability level, in terms of headache intensity and frequency, and the consequent reduced physical activity are often associated with a higher tendency to the symptomatic usage of drugs, which may turn into real medication overuse (6). Moreover, the choice of symptomatic treatments cross-competing for the same metabolic pathway, together with the response unpredictability due to different genotype-phenotype correlations, favors the development of medication overuse and drug resistance in patients (7). Population-based studies report that from 11 to 70% of people with frequent headache present medication overuse, recognized as a factor associated with headache chronification (8) and medication overuse headache (MOH). High intake of symptomatic drugs may lead in the long term to brain white and gray matter modifications (mainly in the periaqueductal gray, thalamus, ventral striatum and multiple frontal and parietal areas) (9, 10) and to increased cortical excitability as well as facilitation of trigeminal nociception (11, 12). Although there is much speculation on these pathophysiological mechanisms, they are still largely unclear. MOH has a wide range of prevalence among adults (0.5–7.2%) and is responsible for high economic costs—e.g., in terms of lost working days—and considerable time/care expenditure (13, 14). In Europe, the drugs most commonly involved in MOH are non-steroidal anti-inflammatory drugs (NSAIDs) (54% of cases), triptans (31%), and to a lesser extent, ergotamines (4%) (15). More than half of patients with MOH (57%) consult general practitioners to obtain information and advice on headache management, while 83% also consult one or more specialists (15). A survey conducted in a large number of patients with headache showed a significant delay, even of several years, in accessing specialized centers, and generally low satisfaction with the therapeutic management of the condition (16). Although a randomized study did not demonstrate a greater therapeutic response in patients followed by a neurologist as opposed to a primary care physician (17), most of the available evidence points in the opposite direction. In a Spanish epidemiological study, up to 60% of patients with CH receiving suitable treatments showed some degree of improvement (18). Moreover, multidisciplinary treatment—performed by physicians, nurses and psychologists—was found to be effective in reducing headache frequency in drug-resistant patients (19). In addition, only about 20% of young people suffering from headache appear to consult a physician to get advice (20), and self-medication in the general population of headache sufferers (44%) (21) may be another possible reason for headache chronification. In this context, the pharmacy might be the first contact point for headache advice (20). In an observational community pharmacy-based study, self-medication was found in 44% of headache sufferers, who mainly used paracetamol (62%), NSAIDs (39%), and combination analgesics (36%). Moreover, 24% overused acute medication, particularly combination analgesics (57%), simple analgesics (45%), and triptans (7%) (21). Although it is reasonable to think that self-medication exposes headache sufferers to a greater risk of overuse, at present, there are no relevant published studies that compare self- and specialist-prescribed medications.

The published evidence on the effect of specialized treatment in outpatients complaining of headache is still limited (22, 23). Therefore, the aim of this study was to evaluate the long-term efficacy of targeted treatment and the possible changes in the clinical picture and quality of life produced by the management of headache by expert specialists.

## Materials and Methods

### Study Design

A sample of 32 pharmacies, representative of the local health district of Pavia (Italy) (inhabitants: 547,926; number of pharmacies: 245), joined the study under the terms of an agreement with the local board of pharmacists. Pharmacies located in the district's urban, rural and hill regions were equally represented. Before the enrollment phase started, pharmacists attended a compulsory three-part theoretical-practical course on headache and appropriate/inappropriate use of symptomatic and preventive drugs (each part lasted 2 h). To recruit headache sufferers for the study, the participating pharmacies' clients were targeted, over a 30 days period, by a press/media campaign entitled “Headache Month.” Headache sufferers wishing to take part were carefully informed by the pharmacists about the project and were invited to answer the following three initial screening questions: “How often do you get a headache?”; “How many analgesics do you take (type and number of doses)?”, “Are you already under the care of a specialist?”. They were also asked to leave their phone number or e-mail address, subject to signing a consent form. A few days later, they were contacted for a telephone interview conducted by a headache expert (IDC) from the C. Mondino Foundation: the users' sociodemographic characteristics were collected, and the features of their headache and drug consumption/overuse were verified. Afterwards, the participants underwent a series of four assessments carried out by two neurologists with specific expertise in headache (FA and IDC): a baseline evaluation (T0), during which they received a diagnosis and therapeutic program, and three follow-up assessments, at 3, 6, and 12 months (T3, T6, and T12, respectively). On each occasion, the participants underwent a complete neurological assessment and received therapeutic adjustments, if necessary, aimed at achieving optimal management of their headache. Moreover, at T0 the participants were given an ad hoc paper headache diary; the diary data collected at T3, T6, and T12 were used for statistical evaluation.

Pain severity was recorded according to a 0–10 numeric rating scale (NRS) (0: no pain, 10: maximum pain ever experienced). Therapeutic response was evaluated in terms of headache frequency, severity, duration, and symptomatic drug consumption. Self-assessment questionnaires, i.e., the Headache Under-Response to Treatment (HURT) (24) and the Migraine Disability Assessment Scale (MIDAS) (25) scales, were also administered to assess disability level and hence the quality of care received.

This study was approved by the local ethics committee. All the participants gave written informed consent for their participation in the study.

### Participants

Each of the participants was a client of one of the local pharmacies involved in the study, who, after receiving detailed information about the study from the pharmacist, filled in the project participation card. The criteria for inclusion in the present study were a history of primary headache (regardless of the frequency and characteristics of attacks), adulthood, willingness, and ability to give written informed consent. There were no particular exclusion criteria apart from the presence of secondary headaches and other chronic pain conditions.

### Outcomes

The primary study outcome was modification of the severity and frequency of the headache attacks and of symptomatic drug consumption, achieved through expert management of the headache. The specialist care was also evaluated for efficacy, in terms of disability reduction, and for perceived quality as assessed using patient self-administered questionnaires. As secondary outcomes of the study we also analyzed reasons for poor compliance and patient dropouts.

### Statistical Analysis

The Kolmogorov-Smirnov test was used to investigate the distribution normality of the different variables and, thus, to choose the correct statistical tests. The Chi-square test (χ2) was used to analyze revisions, at T0, of the initial diagnoses made on the basis of the preliminary telephone interview. Repeated measures ANOVA was used to compare the primary outcome variables across the four time points (T0, T3, T6, and T12). The effects of time on headache features (days/month, attacks/month, attack duration, and severity) and on subjective assessment scales (MIDAS and HURT) were measured using linear regression models. The effects of age, gender, disease duration and headache onset on dropout behavior were evaluated with a logistic regression analysis. The Kruskal-Wallis test was used to detect potential significant differences (in headache days/month, attacks/month, attack duration, attack severity, drug intake) at each time point between dropouts and non-dropouts. All statistical analyses were performed using the R software.

## Results

### Participants' Baseline Features

A total of 272 cards were collected by the pharmacies and sent to the headache specialist to be used for the telephone interview. Of these, 73 were excluded for the following reasons: participant too young (*n* = 8), lack of consent (*n* = 36), incorrect contact details (*n* = 5) and failure to answer the phone call (*n* = 24). Thus, the total number of participants recruited and included in the analysis was 199: 172 women (86%) and 27 men (14%), with a mean age of 45 ± 12 years (62% were in the 40–60 years age range and 30% were aged 20–40 years).

From the data collected during the telephone interview, it emerged that 53% of the sample reported headache onset at 30–40 years of age, and 25% at 20–30 years; 16% presented a form of CH, while the rest of the sample was divided between episodic forms with high (47%) and low (37%) frequency of attacks. Moreover, on the basis of the phone interviews, 126 of the 199 patients experienced five or more days with headache per month (63%) and thus needed preventive therapy.

At the first face-to-face evaluation (T0) most of the patients reported that they used symptomatic drugs: on medical prescription (52%), as self-medication, i.e., over-the-counter drugs (34%) or both (14%), and 28% were already receiving preventive therapy. Moreover, 25% of the patients already presented overuse of symptomatic drugs (analgesics and/or triptans). The headache frequency (days/month) and monthly symptomatic drug consumption declared during the telephone interview were generally confirmed at the first specialist assessment (11 ± 9 vs. 10 ± 9, *p* = 0.281; 10 ± 12 vs. 12 ± 16, *p* = 0.856, respectively).

### Effects of Specialist Care

The diagnoses made on the basis of the telephone interview and those formulated after the first face-to-face assessment differed significantly, particularly with regard to migraine without aura and cluster headache (Table 1). Indeed, the clinical re-assessment at T0 led to a significant revision of the telephone-based diagnoses (*p* < 0.001) and to diagnosis of the previously unspecified forms.

**Table 1:** Diagnoses formulated on the basis of telephone interview and after the baseline assessment (T0).

**Diagnosis**	**Telephone interview (*N* = 199)**	T0 (*N* = 179)^*^
Migraine without aura	48	161
Migraine with aura	10	6
Tension-type headache	14	10
Cluster headache	9	1
Trigeminal neuralgia	1	0
Aura without migraine	2	0
Unspecified/Other	115	1

* *The group at T0 is smaller as 20 individuals dropped out*.

**Figure 1:**
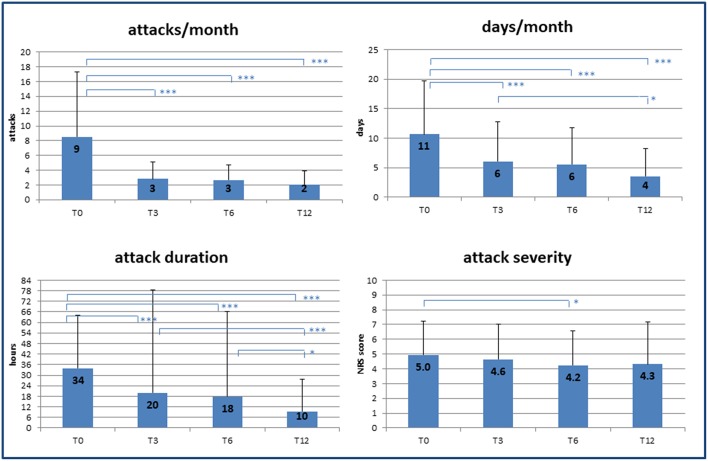
Effect of specialist care on headache during follow-up. Changes in frequency and severity of headache after care was taken over by medical experts (headache specialists). T0, T3, T6, T12 = baseline (T0) and follow-up time points (months). NRS, numerical rating scale for pain assessment. *p*-value * < 0.05, ** < 0.01, *** < 0.001.

Over the 12 months follow-up, we observed a significant decrease in all the variables considered as primary outcomes of the study (Figure 1). In particular, at 3 months significant decreases were already detected in the frequency of attacks (T0: 9 ± 9/month vs. T3: 3 ± 2/month; *p* < 0.001) and in headache days/month (T0: 11 ± 9 vs. T3: 6 ± 6; *p* < 0.001). Single attack duration was also already decreased at the first follow-up appointment (T0: 34 ± 30 h vs. T3: 20 ± 58 h; *p* < 0.001), while pain intensity remained unchanged over time except for a small but significant reduction at T3 (T0: 5.0 ± 2.3 vs. T3: 4.2 ± 2.9 NRS; *p* = 0.038). Analysis with linear regression models confirmed a significant effect of time on the reduction of both headache load and related disability (*p* < 0.001).

**Figure 2:**
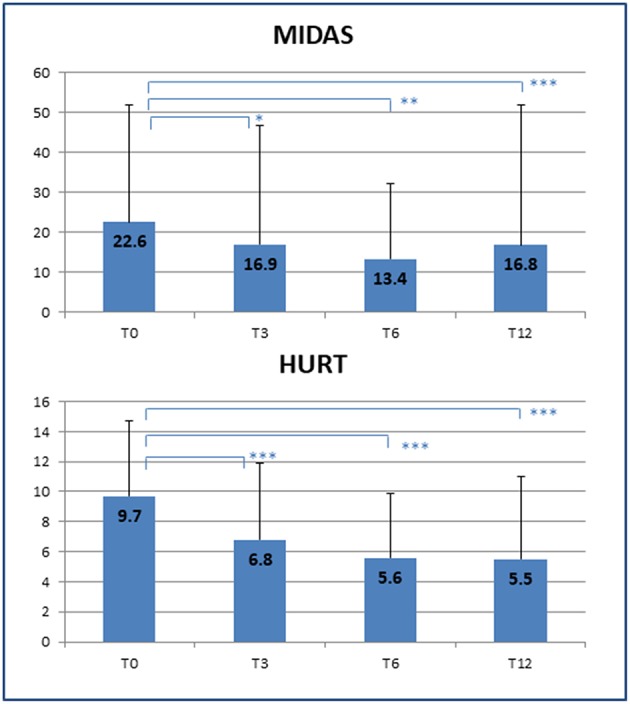
Improvement of quality of life. Decrease in disability scores at the end of the clinical follow-up. T0, T3, T6, T12 = baseline (T0) and follow-up time points (months). MIDAS, Migraine Disability Assessment Scale; HURT, Headache Under-Response to Treatment. *p*-value * < 0.05, ** < 0.01, *** < 0.001.

The improvement in headache management resulted in a significant decrease in symptomatic drug consumption (T0: 12 ± 16 vs. T12: 4 ± 6 doses/month; *p* = 0.014) as well as an increased quality of life, as shown by a reduction in MIDAS scores (T0: 22.6 ± 29.3 vs. T12: 16.8 ± 35.1; *p* < 0.001), and better patient-rated quality of the received care, as assessed by HURT (T0: 9.7 ± 5.0 vs. T12: 5.5 ± 5.5; *p* < 0.001) (Figure 2). Unfortunately, the number of MIDAS and HURT questionnaires administered was not sufficient to allow assessment of any statistical effect of variables such as gender, age, and duration of illness on the improvement of quality of life.

**Figure 3:**
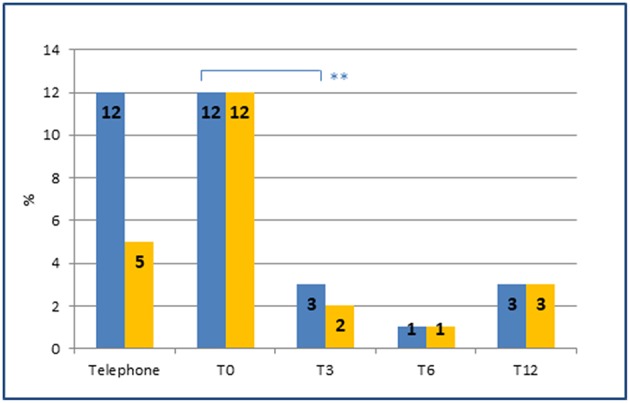
Prevalence of MOH and of preventive therapies at each time point. Prevalence of MOH (blue) and MOH in preventive therapy (orange) at each time point. Note the sharp reduction of MOH (from 12 to 3%) after the introduction of an appropriate preventive therapy by the headache specialist at T0 (from 5 to 12%). **represents the significance level. *p*-value < 0.01.

Finally, the introduction of an appropriate preventive therapy by a specialist led, as early as the 3 months follow-up, to a significant reduction in the number of patients with MOH (*p* = 0.006) (Figure 3).

### Dropouts

**Figure 4:**
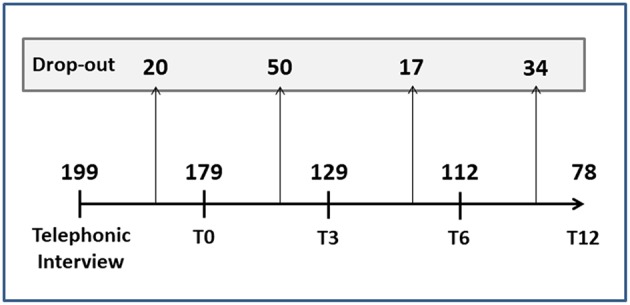
Drop-outs during the follow-up period. T0, T3, T6, T12 = follow-up time points (months).

Figure 4 reports the number of dropouts (tot = 121, 61%) at the different follow-up time points. Most of the patients who abandoned the study had migraine with aura or tension-type headache, while, as expected, the patients with MOH unresponsive to preventive therapy remained in the study until T12. Age, gender, disease duration, age at onset, and geographic origin did not significantly affect dropouts. At all the time points, the participants choosing to drop out of the study were characterized by frequent attacks and several days/months with headache. No significant differences in duration and intensity of attacks were found between the dropouts and the non-dropouts.

## Discussion

In this study, we assessed the effect of expert headache management on headache attack severity and frequency and on symptomatic drug consumption in patients previously self-medicated or cared for by their general physician. Previous studies have investigated this issue, but in small populations and only in patients suffering from MOH; moreover, their participants were mainly enrolled among patients of general physicians or from headache centers (17, 19). This may have constituted an important selection bias, since patients who seek medical attention are more likely than the general population to suffer from more severe and disabling headache attacks. For this reason, community pharmacy-based studies are to be considered more suitable for investigations like ours, as the study populations are more representative of the real headache patterns in the community (26, 27). Moreover, in our study, pharmacists received specific instruction on the topics of headache and appropriate/inappropriate use of symptomatic drugs. This allowed us to collect patients prone to self-diagnosis and self-medication (28), previously unaware of the severity of their condition and of their need for specialist support. Indeed, while 90% of the interviewed subjects were aware of the existence of headache centers, only half (49%) had used them at least once. Furthermore, consistently with what was reported in a recent large observational study in Belgium (44%) (21), 33% of our sample had never consulted a doctor and were regularly practicing self-medication.

The headache management provided by the specialists resulted in a significant improvement in the frequency, duration and severity of attacks, and hence in a lower disease burden for the patients. This observation is consistent with previous studies suggesting that targeted therapy and a multidisciplinary treatment guided by a specialist are effective in treatment-resistant patients (18, 19). In particular, the ability of the team of experts to recognize and manage behavioral superstructures that can complicate the clinical profile greatly increases the efficacy of the rehabilitation program (29). In our study, a significant response to the new treatment was observed from as early as the first follow-up appointment, supporting the view that management by a specialist has its greatest impact early on in the patient's care. Probably, the more accurate diagnosis furnished by the specialist plays a decisive role in this respect. Indeed, in non-clinic populations, headache diagnosis is often delayed and requires repeated medical consultations (with a mean of 4.3 physicians) before the condition is correctly defined (30, 31). This, in turn, generates treatment mistakes, often resulting in therapeutic ineffectiveness. Moreover, general physicians are still not well acquainted with preventive treatments: more than a third of them do not prescribe preventive therapy in situations in which, according to the current guidelines, it is necessary (31). In the American population, one in four migraineurs would be eligible for preventive therapy, but only 13% of patients receive an appropriate drug prescription (32). In our study, of the 126 patients needing headache prophylaxis, only 28% actually received it.

Among the 199 interviewed patients, 25% presented medication overuse, involving analgesics in 60% and triptans in 40% of cases, while 12% met the diagnostic criteria for MOH. In the general population, the prevalence of MOH ranges from 0.5 to 7.2% (33–35), while higher rates are reported among patients attending headache centers, i.e., 25% in Europe (36) and 60% in United States (37, 38). As expected, MOH prevalence in our community pharmacy-based sample was close to (only slightly higher than) that of the general population: the slightly higher prevalence in our sample can be attributed to a selection bias due to the enrollment setting (symptomatic patients in a drug store). For similar reasons, the proportion of analgesics (15%) and triptans (10%) overusers in our sample is higher than that reported in previous population-based studies (39). The ratio between analgesic and triptan overuse is also consistent with data from the literature (15, 21). The introduction of a targeted preventive therapy led, in both drug groups (analgesic and triptan overusers), to a sharp decrease in monthly drug intake, already observable at 3 months of follow-up. This is in agreement with previous observations on the efficacy of the specialist approach in patients with chronic and overuse headache (18, 19).

Reasons leading patients to abandon therapeutic follow-up are still poorly known. A study in patients with cluster headache identified prolonged clinical remission as a major determinant of dropout status (40). In our sample, no significant differences in terms of disease severity were found between the dropouts and the participants who remained in the follow-up program. Therefore, there is no evidence to suggest that the participants who chose to forego further specialist follow-ups did so as a result of dramatic changes in their condition (whether it improved or worsened). Moreover, it is interesting to note that geographic origin (urban vs. rural centers) did not influence the percentage of dropouts during the follow-up: the same therapeutic management was found to be equally effective across all the geographical locations.

Specialist intervention resulted in a significant reduction of the impact of headache on daily quality of life (assessed with the MIDAS scale) from as early as the first follow-up assessment. At the same time point, the participants felt the treatment to be more effective (as shown by the results on the HURT scale). This is consistent with the results of a previous study in European headache centers, which reported an increase in subjectively perceived effectiveness in 70% of patients followed by a specialist (22).

In conclusion, the findings of this study suggest that headache management by a specialist is more effective than self-treatment, in particular in chronic headache or MOH patients, resulting in an overall benefit. This further supports the clinical utility of a network involving headache specialists, general physicians and trained pharmacists, which could detect individuals who might benefit from targeted multidisciplinary treatment provided by headache centers. From this point of view, the pharmacist may act as a first-line health provider in the community for individuals in need of specialist attention. Future studies should focus on potential differences of an interregional or intercultural (e.g., North vs. South) nature, and on differences between different countries.

## Data Availability

The datasets generated for this study are available on request to the corresponding author.

## Ethics Statement

This study was approved by the local ethics committee. All the participants gave their written informed consent to their participation in the study.

## Contribution to the Field

Headache is a common pain condition, responsible for varying degrees of disability. Its chronic forms, in particular, are associated with high levels of healthcare demand and drug consumption. Moreover, high intake of symptomatic drugs may promote nociception facilitation mechanisms that lead to the development of MOH. In this scenario, self-medication is an important risk factor for overuse, especially among patients not previously evaluated by a physician. This study shows that management by specialists in headache centers is more effective than self-treatment, particularly in chronic or MOH patients, resulting in an overall benefit, both in terms of disability and the need for symptomatic drugs. Our findings support the clinical utility of a network involving headache specialists, general physicians and trained pharmacists, which could detect individuals who might benefit from targeted multidisciplinary treatment. In this scenario, the pharmacist may act as a first-line health provider in the community for individuals in need of specialist attention.

## Author Contributions

FA: study concept and design. FA and ID: clinical consultant and data collection. MC: statistical analysis and interpretation of data. MC and FA: drafting of the manuscript. FA and AC: critical revision of the manuscript.

### Conflict of Interest Statement

The authors declare that the research was conducted in the absence of any commercial or financial relationships that could be construed as a potential conflict of interest.
